# NIH PATHWAYS TO PREVENTION WORKSHOP: RESEARCH GAPS FOR LONG-TERM DRUG THERAPIES FOR FRACTURE PREVENTION

**DOI:** 10.1093/geroni/igz038.2743

**Published:** 2019-11-08

**Authors:** Lyndon Joseph, Faye Chen

**Affiliations:** 1 National Institute on Aging, Bethesda, Maryland, United States; 2 NIAMS/NIH, Bethesda, Maryland, United States

## Abstract

The NIH Pathways to Prevention Workshop was held on October 30-31, 2018 to present scientific evidence, as well as physician and patient perspectives to better understand the benefits and harms of drug therapies for osteoporosis fracture prevention. Osteoporotic fractures lead to substantial morbidity, mortality, and economic costs. The underlying medical condition, is a skeletal disorder characterized by compromised bone strength predisposing to increased fracture risk. Several medications approved by the U.S. Food and Drug Administration to prevent osteoporotic fractures have been effective when taken by people who are at high risk of fracture. These include bisphosphonates , denosumab, teriparatide, estrogens, and selective estrogen-receptor modulators. However, rare but serious adverse events, such as atypical femoral fractures and osteonecrosis of the jaw associated with bisphosphonates have raised questions regarding the safety of their use. There is limited evidence on the benefits and harms of long-term osteoporosis drug therapy, including the timing and duration of drug discontinuation or drug holidays. It is not clearly known which patients will benefit or may be harmed from continued drug intervention, who should and should not be put on drug holiday, how to identify such patients and properly treat them, and how to predict their outcomes. Key scientific areas covered by the workshop included: the benefits and risks of osteoporotic drugs with short-term and long-term use and factors that influence outcomes; the impact of drug discontinuation and drug holidays on outcomes; and patient and clinician factors that impact the use of and adherence to osteoporotic drugs.

